# Research on non-tobacco related materials recognition method based on YOLOv8

**DOI:** 10.1038/s41598-025-19653-9

**Published:** 2025-10-13

**Authors:** Chunjie Zhang, Lijun Yun, Mingjie Wu, Ruilin Luo, Zaiqing Chen, Feiyan Cheng

**Affiliations:** 1 Yunnan Normal University, Kunming, 650000 China; 2 Engineering Research Center of Computer Vision and Intelligent Control Technology, Department of Education of Yunnan Province, Kunming, 650000 China; 3 Equipment Information Department of Yunnan Tobacco and Leaf Company, Kunming, 650218 China

**Keywords:** NTRM-YOLO, Non-tobacco related materials, Object detection, YOLOv8, Deep learning, Computer science, Electrical and electronic engineering

## Abstract

Enhancing non-tobacco related materials control and improving the purity of tobacco leaves have emerged as pivotal quality indicators for raw material processing in both domestic and foreign industrial enterprises. In order to accurately detect non-tobacco related materials, this paper introduces an enhanced variant of the YOLOv8(You Only Look Once version 8) model, termed NTRM-YOLO. NTRM-YOLO use deep learning methods to detect non-tobacco related materials. The attention mechanism module is integrated into the backbone network of NTRM-YOLO, aimed at enhancing the delineation of non-tobacco related materials features, thereby bolstering the detection efficacy of the model. In order to reduce the number of model parameters, this paper integrates GhostConv(Ghost Convolution) module within the neck network, alongside the design integration of a GhostConv-C2F module. This strategic substitution serves to diminish the model’s parameters while concurrently enhancing its capacity for feature expression. Within the Head network, capitalizing fully on the merits of multiple attention mechanisms, Dyhead(Dynamic Head) is introduced with the aim of markedly enhancing the detection accuracy of the model. This study also optimized the loss function by using the vector angle. Moreover, this paper uses industrial camera sensors to collect images containing non-tobacco related materials and constructed of an NTRM dataset after preprocessing. Subsequently, a meticulously series of experiments was conducted on the NTRM dataset to showcase the efficacy of NTRM-YOLO model in applications pertaining to non-tobacco related materials detection. The experimental findings reveal that in contrast to the baseline model, NTRM-YOLO attained a detection performance of 95.6%, marking a notable improvement of 2% over the baseline model. Additionally, it exhibited a parameters of 10.0 MB, reflecting a 10% reduction compared to the baseline model. These experiments furnishes a theoretical foundation and technical substantiation for the subsequent advancement of more refined industrial impurity removal instruments and equipment.

## Introduction

Given the escalating global demand for tobacco safety, cigarette enterprises are increasingly focusing on non-tobacco related materials control^1^. Prior to being transformed into cigarettes, tobacco leaves must undergo numerous stages and processes including harvesting, curing, grading and redrying^2^. Non-tobacco related materials may mixed in each of these steps, posing significant challenges to production and processing and impacting the final cigarette quality. The inclusion of non-tobacco related materials, such as stones, metal, glass, etc., in production and processing equipment can lead to the easy damage of these equipment. The incorporation of non-tobacco related materials into cigarette products can alter the smoking experience and adversely affect cigarette brands. In severe instances, the combustion of chemical products such as plastic and rubber can generate toxic gases, posing risks to consumers and significantly impacting public health. To guarantee the safety of cigarette consumption, the tobacco industry maintains a zero-tolerance policy towards non-tobacco related materials within tobacco leaves, necessitating their thorough removal to ensure tobacco leaves safety.

Currently, industrial enterprises primarily depend on manual impurity removal to pick up the non-tobacco related materials^3^. Within the tobacco sorting production line, sorting workers undertake the task of removing impurities from the tobacco. Positioned at intervals of less than one meter along both sides of the conveyor belt, these workers meticulously and attentively sort through non-tobacco related materials. This process entails significant time and energy expenditure, with strenuous tasks and a substantial workload. Throughout the annual tobacco sorting season, a considerable number of sorting workers are necessitated to handle the sorting of non-tobacco related materials. Nevertheless, this sorting method hinges solely upon human subjective judgment, non-tobacco related materials, like paper scarps, which significantly contrast with tobacco leaves, are readily discerned. However, distinguishing certain non-tobacco related materials, such as yellowish weeds, which closely resemble yellow tobacco leaves, proves challenging to the naked eye, often resulting in oversight during the selection process. The substitution of manual labor with automation has emerged as a prevailing trend, and the advancement of impurity removal robot holds promise in enhancing the efficiency of non-tobacco related materials extraction, alleviating labor demands, and mitigating labor expenses. However, realizing this objective necessitates comprehensive research on robotic for impurity removal. Primarily, there is a requisite for the development of a suite of automated and intelligent methodologies to precisely detect non-tobacco related materials.

While numerous methods for object detection are already exist^4–6^ there lacks a dedicated detection methodology tailored for non-tobacco related materials. The detection and removal of non-tobacco related materials occur within industrial production lines, so the algorithms need to fulfill not only real-time industry demands but also uphold high accuracy. In the field of object detection, the YOLO algorithm has developed rapidly since its inception due to its fast and accurate characteristics^7^. To ensure the quality of tobacco leaves, this study opted to employ the YOLO algorithm for the detection of non-tobacco related materials. However, due to the diverse types of non-tobacco related materials, some of which are very similar to tobacco leaves and can be easily obscured by the leaves, the existing YOLO algorithm is not satisfactory in detecting non-tobacco related materials. This study has refined the YOLOv8s and proposed NTRM-YOLO to identify non-tobacco related materials mixed in tobacco leaves, thereby enhancing the purity of the tobacco leaves.

To detect non-tobacco related materials, it is necessary to have a non-tobacco related materials dataset that includes images and annotations of non-tobacco related materials. However, there is currently no publicly accessible dataset pertaining to non-tobacco related materials. Therefore, this study used a self-build dataset of non-tobacco related materials. By using an industrial camera sensors to capture images of tobacco leaves, preprocessing them, and using annotation tools to annotate the non-tobacco related materials contained within them, then randomly dividing them into training and test sets, the final non-tobacco related materials dataset used in this study is formed, called NTRM dataset.

Overall, this study mainly focused on:

1. Constructed the NTRM dataset. Collecting images of tobacco leaves through industrial camera sensors, a total of over 3000 images were collected. Each sample encompasses not only tobacco leaves but also diverse quantities and types of non-tobacco related materials. In order to conserve both time and labor costs, this study employs a semi-automatic labeling method for the annotation of non-tobacco related materials.
2. NTRM-YOLO model was introduced for the detection of non-tobacco related materials. Introducing the attention mechanism module into the backbone network to enhance feature extraction capabilities, facilitating the extraction of features pertaining to non-tobacco related materials. GhostConv is adopted and introduced into C2F to form the GhostConv-C2F module, thereby reducing model parameters and enhancing feature expression capabilities. Optimizing detection heads in the head layer and incorporating the Dyhead module. Finally, the loss function is optimized to enhance the detection accuracy and improve the identification accuracy of non-tobacco related materials.
3. A meticulously designed series of experiments was conducted to validate the efficacy of the model on the NTRM dataset. The experimental outcomes revealed that NTRM-YOLO can effectively detect non-tobacco related materials.

## Related work

### Dataset construction

With the rapid evolution of deep learning, a multitude of associated research algorithms persistently emerge. Various datasets are required to train models for different scenarios, resulting in the proliferation of datasets. Currently, there are two types of datasets: One category comprises openly accessible public datasets available on the internet, such as the renowned COCO^8^ VOC^9^ and VisDrone^10^ datasets. Additionally, in different application fields, there are also public datasets, including the UC Merced^11^ WHU-RS19^12^ and AID^13^ datasets for remote sensing imagery, human behavior recognition dataset KTH^14^ Weizmann^15^. The second category is also gaining traction among teams, as datasets are constructed based on specific requirements, exemplified by the photovoltaic panel IR fault dataset^16^ cherries dataset^17^ SlamlightClass dataset^18^ underwater feed pellets dataset^19^ and tea diseases and insect pests dataset^20^. The investigation of non-tobacco related materials, as explored in this article, is tailored to specific requirements. Currently, there is an absence of relevant public datasets accessible online. Consequently, this study collected samples of non-tobacco related materials and established a NTRM dataset.

For sample collection, it is typically involves online crawling or using camera sensors for photography. However, the internet offers a limited quantity of images depicting non-tobacco related materials. Therefore, this study opted to utilize camera sensors for sample collection. Lu et al. devised an image acquisition system tailored to tobacco grading. This system incorporates a light source and a sealed device, employing a GIG2630 camera to vertically capture images of samples positioned on the white sample platform below^21^. Laval uses a digital commercial camera to capture images of tomato plants under natural Light, with a distance of 0.5–1.0 m between the camera sensor and the tomato plants in the field^22^. Wang et al. designed an image capture darkroom and gathered four types of tobacco shreds employing an industrial camera^23^. Owing to their brief shutter duration, industrial cameras can capture moving objects while maintaining high image quality. In this investigation, industrial camera sensors were deployed to capture images of non-tobacco related materials. Secure the industrial camera sensor onto a bracket positioned directly above the conveyor belt, ensuring a frontal view of the tobacco sample as it moves along the belt. Maintain consistent height and angle of the industrial camera sensor throughout the entire capturing process.

### Object detection

With the rapid development of deep learning in recent years, object detection based on deep learning has also made significant advancements^24,25^. Deep learning-based object detection typically falls into two distinct categories^26^. The two-stage algorithm requires extracting candidate boxes before detection, resulting in a slower process, whereas the one-stage algorithm directly detects objects, resulting in a faster detect. For the detection of non-tobacco related materials, it needs to be directly carried out on the production line, so a real-time algorithm is needed. This study chose to use a one-stage algorithm. Currently, the YOLO series algorithm^27–35^ represent a highly popular and efficient selection within the realm of one-stage methodologies. YOLO has found widespread application across various industries, including autonomous driving, video surveillance, and medical detection^36^ demonstrating commendable performance in detecting relevant targets. Notably, it satisfies real-time demands while maintaining a high level of accuracy. This study elected to enhance YOLO for the detection of non-tobacco related materials.

In the tobacco industry, some researchers have also applied the YOLO algorithm. Lin et al. improved YOLOX-tiny to detect tobacco brown spot disease^37^. Nasir et al. used YOLOv5n to detect tobacco crop^38^. Park et al. improved YOLOv4-tiny to detect packaging defects in cigarette^39^. However, research on the detection of non-tobacco related materials is presently limited. Li and Huang et al. utilized hyperspectral imaging technology alongside machine learning methods to classify and identify non-tobacco related materials in tobacco leaves. However, the cost associated with hyperspectral imaging remains relatively high. This study introduces the NTRM-YOLO algorithm designed for detecting non-tobacco related materials under visible light conditions. Based on YOLOv8, the algorithm integrates CPCA(Channel Prior Convolutional Attention) module into the backbone to enhance feature extraction capabilities. Furthermore, computational complexity is mitigated within the neck layer, and optimization of the detection head and loss function is undertaken to bolster both detection accuracy and inference speed. Experimental validation is conducted using the NTRM dataset, affirming its effectiveness.

## NTRM dataset

### Data acquisition

**Fig. 1:**
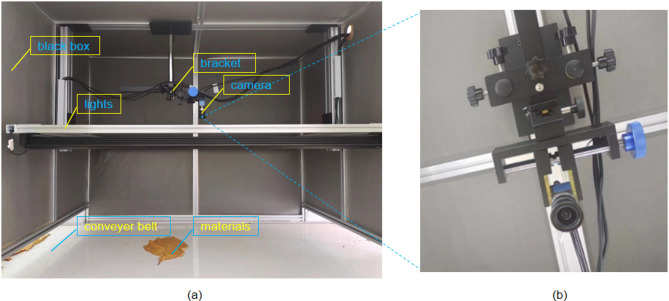
Image acquisition equipments.

Due to the lack of existing open-source datasets for non-tobacco related materials, we collected images of tobacco and non-tobacco related materials. The samples of tobacco and non-tobacco related materials utilized in this study were provided by yunnan tobacco leaf company. In order not to affect actual production, this study first simulated a production line in the laboratory, collected experimental data, trained the model, and then applied it to actual production. The collection devices is shown in the Fig. 1.

The image acquisition system mainly consists of conveyor belt, industrial camera, light sources, darkbox, bracket, etc., as shown in Fig. 1(a). The industrial camera utilizes the MER-G-P digital camera for data collection. The height of the camera can be adjusted through the bracket. In this study, the distance between the industrial camera and the conveyor belt was fixed at 0.8 m, located directly above the conveyor belt, as shown in Fig. 1(b). During the acquisition process, maintain the position and parameters of the camera.

When starting the shooting, start the conveyor belt and place the materials on it. The industrial camera will automatically take pictures. We use a collection system to automatically save the images. Considering the different data environments in real application scenarios, we collected several types of images, such as: no non-tobacco related materials, one non-tobacco related materials, multiple non-tobacco related materials, etc. A total of 3160 images were collected, with a single image size of 2048 * 2248 pixels, saved in JPEG format. Each image has three spectral channels, R, G, and B.

**Fig. 2:**
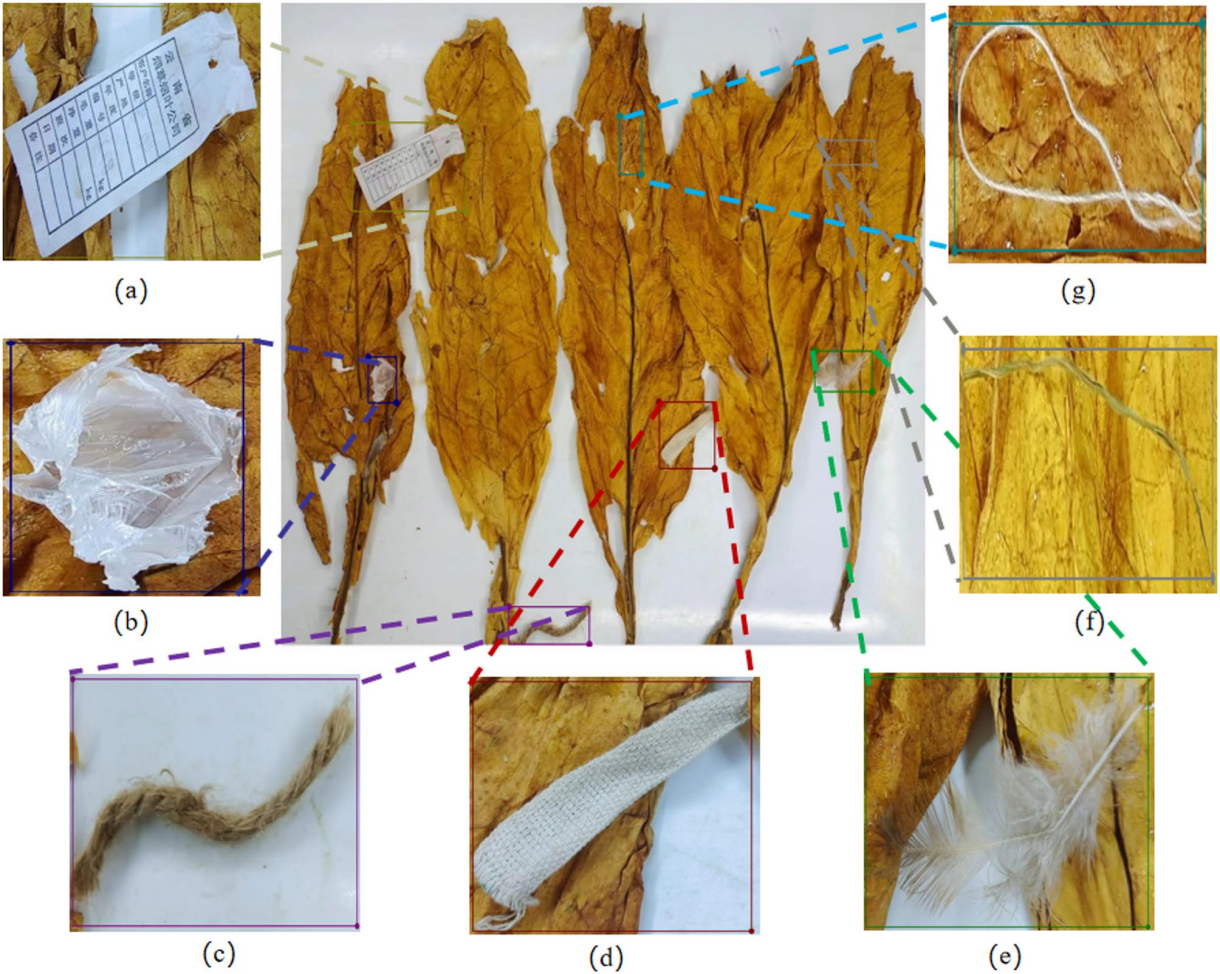
Samples of non-tobacco related materials. (**a**) paper scrap; (**b**) plastic; (**c**) hemp rope; (**d**) cloth strip; (**e**) feather; (**f**) weed; (**g**) fine-line.

This study gathered a total of seven common miscellaneous categories found in tobacco production environments, varying in colors and materials. These include cloth strip, plastic, paper scrap, fine line, feather, weed and hem rope. An example of non-tobacco related materials is illustrated in Fig. 2.

### Semi-automated data annotation

LabelImg was utilized to annotate the non-tobacco related materials within the samples and save the annotations in YOLO format (class, x, y, width, height) as a TXT file. Nevertheless, because there are too many non-tobacco related material instances, manual annotation proves to be time-consuming, labor-intensive, and susceptible to errors. Therefore, this study employs a semi-automatic data annotation method.

**Fig. 3:**
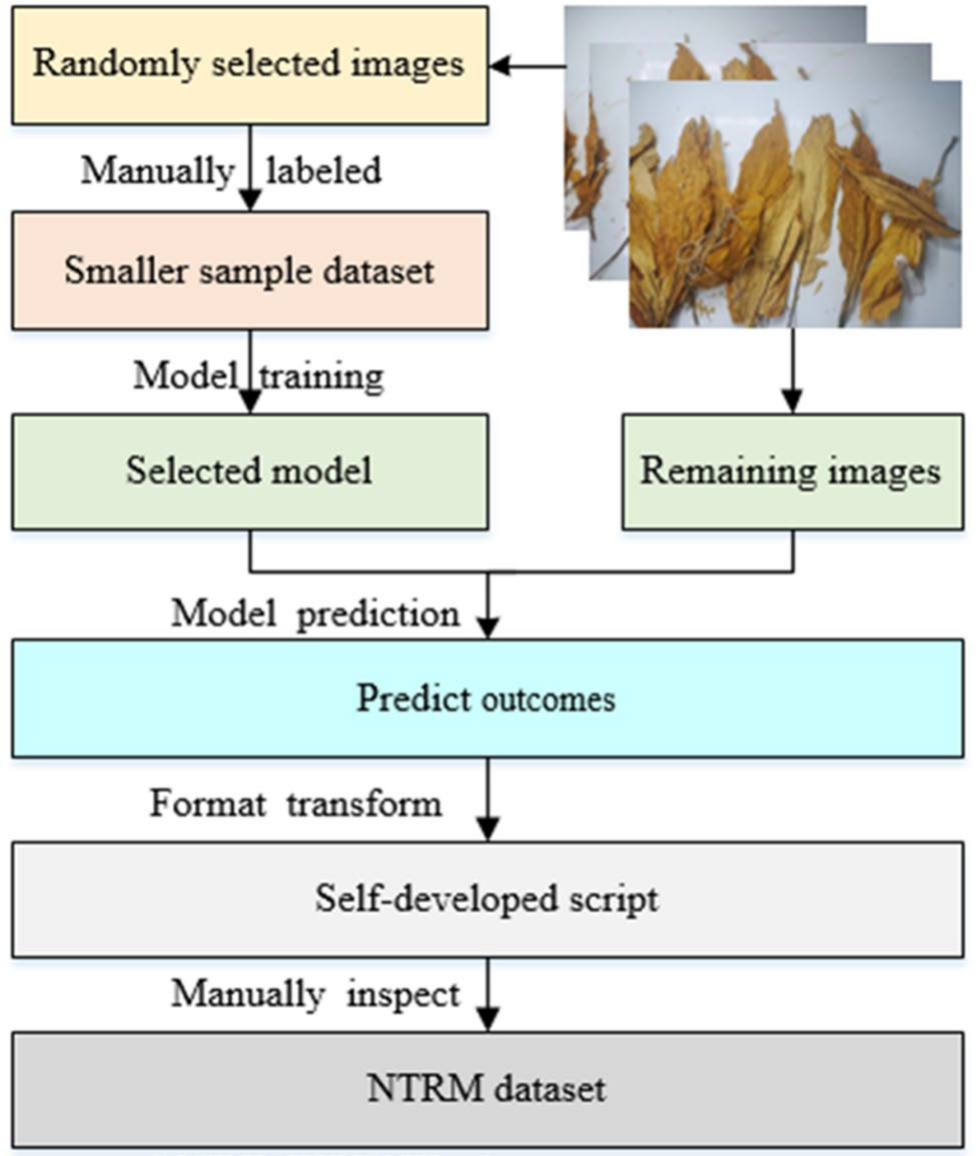
Semi-automated annotation data process.

Initially, a small sample dataset consisting of 100 randomly selected images was chosen, and the non-tobacco related materials within these images were manually labeled using LabelImg. Secondly, employing the YOLOv8s model for training with 100 epochs facilitates expedited model training on the small sample datasets. Following training, the model demonstrating the most favorable training outcomes is selected. Subsequently, the selected model is employed to predict the remaining images, and a self-developed script is utilized to transform the predicted outcomes into a YOLO format. Lastly, import the predicted outcomes into LabelImg, manually inspect them, and rectify any inaccuracies in the predictions. Inspect the annotations meticulously to mitigate the risk of omission or mislabeling. Subsequently, partition the annotated samples in a 8:2 ratio and randomly divide them into training and test sets, form the NTRM dataset. The process of semi-automatic annotation is shown in Fig. 3.

The NTRM dataset comprises a total of 3160 images, with the training set consisting of 2528 samples and the test set consisting of 362 samples. Within the training set, there are a total of 7779 instances, while the test set comprises a total of 1942 instances.

### Data enhancement

Given the limited dataset and the disparate distribution of non-tobacco materials across various categories, this study sought to mitigate the risk of overfitting by augmenting the training data. There are several methods to augment the dataset^40^ and this study adopts techniques such as horizontal flipping, vertical flipping, and random cropping to enrich the training set. To expedite this process and conserve human resources, custom conversion scripts were employed to convert the annotations of the added samples.

## NTRM-YOLO model

### Overall architecture

**Fig. 4:**
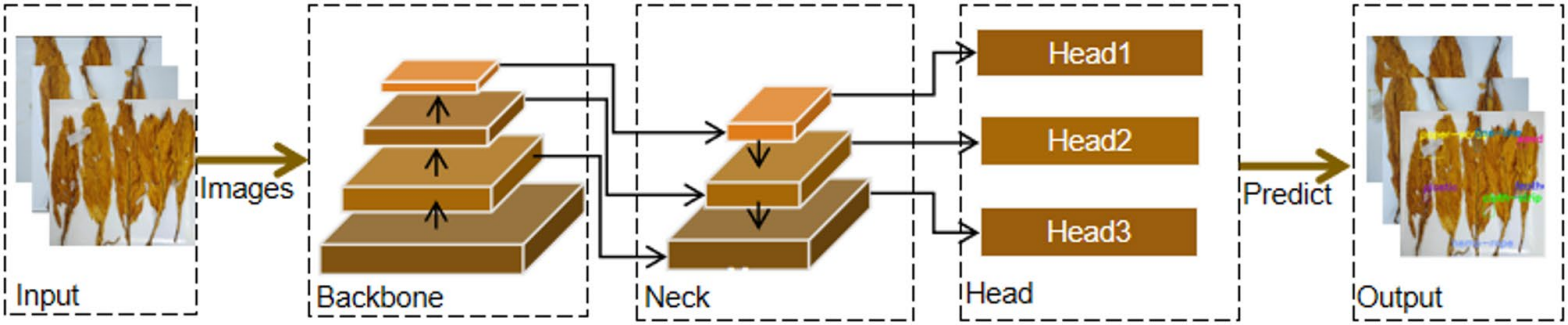
The structure of NTRM-YOLO.

YOLOv8 includes YOLOv8n, YOLOv8s, YOLOv8m, YOLOv8l, and YOLOv8x due to different depths and widths. Given that the model discussed in this article necessitates deployment within the production line of the tobacco sorting workshop, it is advisable to avoid selecting a model that is excessively large. YOLOv8s offers the benefits of both high accuracy and a compact model size, in this paper, the YOLOv8s network is chosen as the baseline model. We propose NTRM-YOLO, which is based on the baseline, with its structure depicted in Fig. 4.

The NTRM-YOLO detection network consists of four parts:

Input is responsible for the input of the model. During model training, images and labels in the NTRM training set are input, while during model testing, images and labels in the NTRM test set are input.

Backbone serves primarily for feature extraction, comprising modules such as Conv, C2F, and SPPF. This investigation introduces the CPCA module, designed to focuses on important features in both channel and spatial dimensions. By dynamically adjusting weights, it mitigates recognition errors arising from the resemblance between non-tobacco related materials and tobacco leaves, thereby improving the accuracy of non-tobacco related materials identification.

Neck is to fuse multi-scale features. In this analysis, GhostConv is introduced and GhostConv-C2F is designed to curtail model parameters while enhancing the representational capacity of feature information.

Head utilizes the obtained features for prediction. In this study, Dyhead is introduced to reduce design complexity, improve position accuracy and model generalization ability, making it more flexible. By using three different size feature maps to facilitates the acquisition of both category and positional information, the detection results of non-tobacco related materials are ultimately obtained.

### The backbone network of NTRM-YOLO

**Fig. 5:**
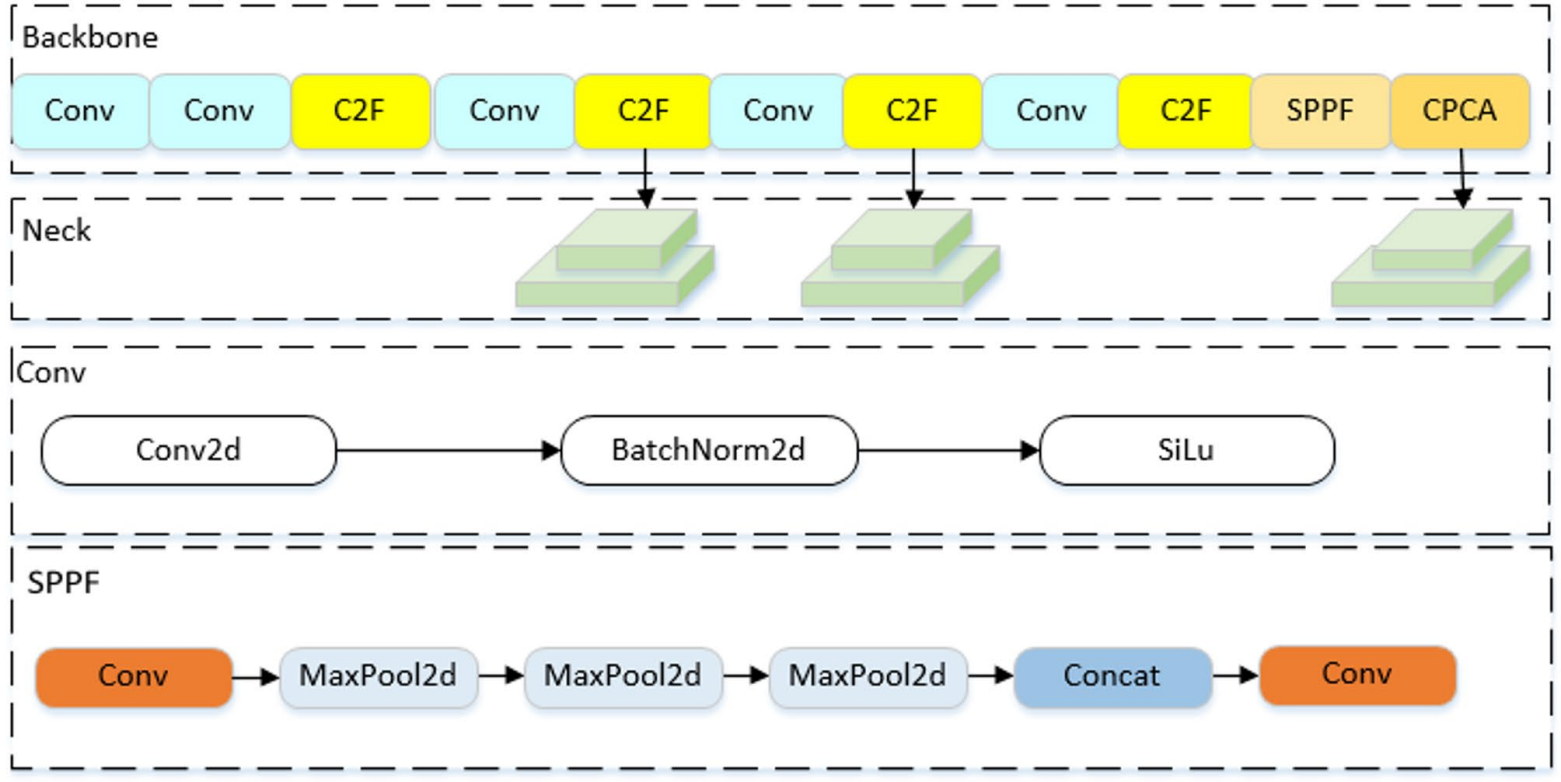
The backbone network of NTRM-YOLO.

Researchers emulate the selective perception mechanism of the human visual system and propose attention mechanisms. The purpose is to direct the model’s focus towards salient regions of features, and ignore unimportant ones. In this research, we introduced the CPCA module into the backbone network of the baseline model, located after SPPF, to form the new backbone of NTRM-YOLO, as shown in Fig. 5.

**Fig. 6:**
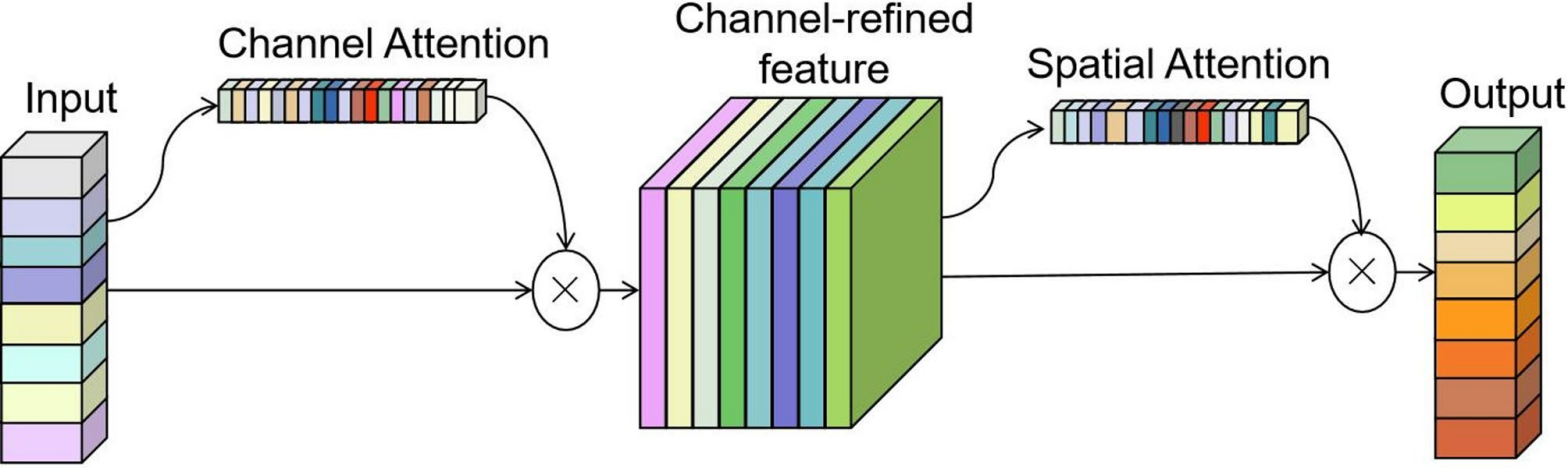
The structure of CPCA module.

CPCA^41^ module can simultaneously obtain spatial information and channel information, this augmentation aids the model in more accurately discerning the characteristics of non-tobacco related materials objects. CPCA utilizes deep-wise convolutional modules to construct spatial attention mechanism, ensuring effective information extraction while reducing computational complexity. The structure of CPCA is shown in Fig. 6.

**Fig. 7:**
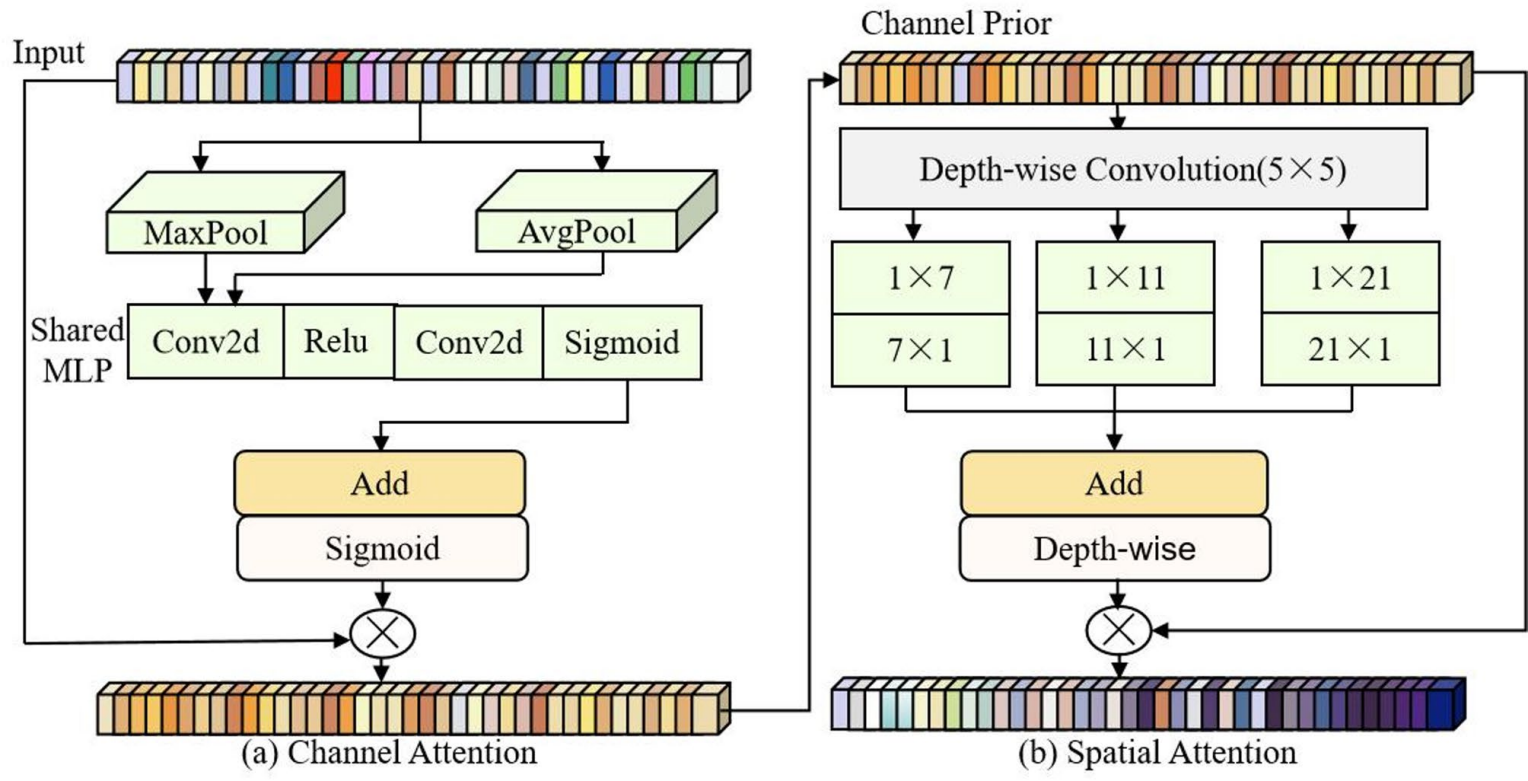
The channel and spatial attention of CPCA.

CPCA performs channel attention and spatial attention in sequence. Channel attention consolidates spatial information from feature maps through max pooling and average pooling operations. Subsequently, this information undergoes processing via a Shared MLP, followed by summation, and then multiplication with input features to generate channel priors (CP). Subsequently, the CP is input into the deep convolution module to generate a spatial attention map (SAM). Finally, the output of CPCA is obtained by element wise multiplication of CP and SAM, as shown in Fig. 7.

### The neck network of NTRM-YOLO

**Fig. 8:**
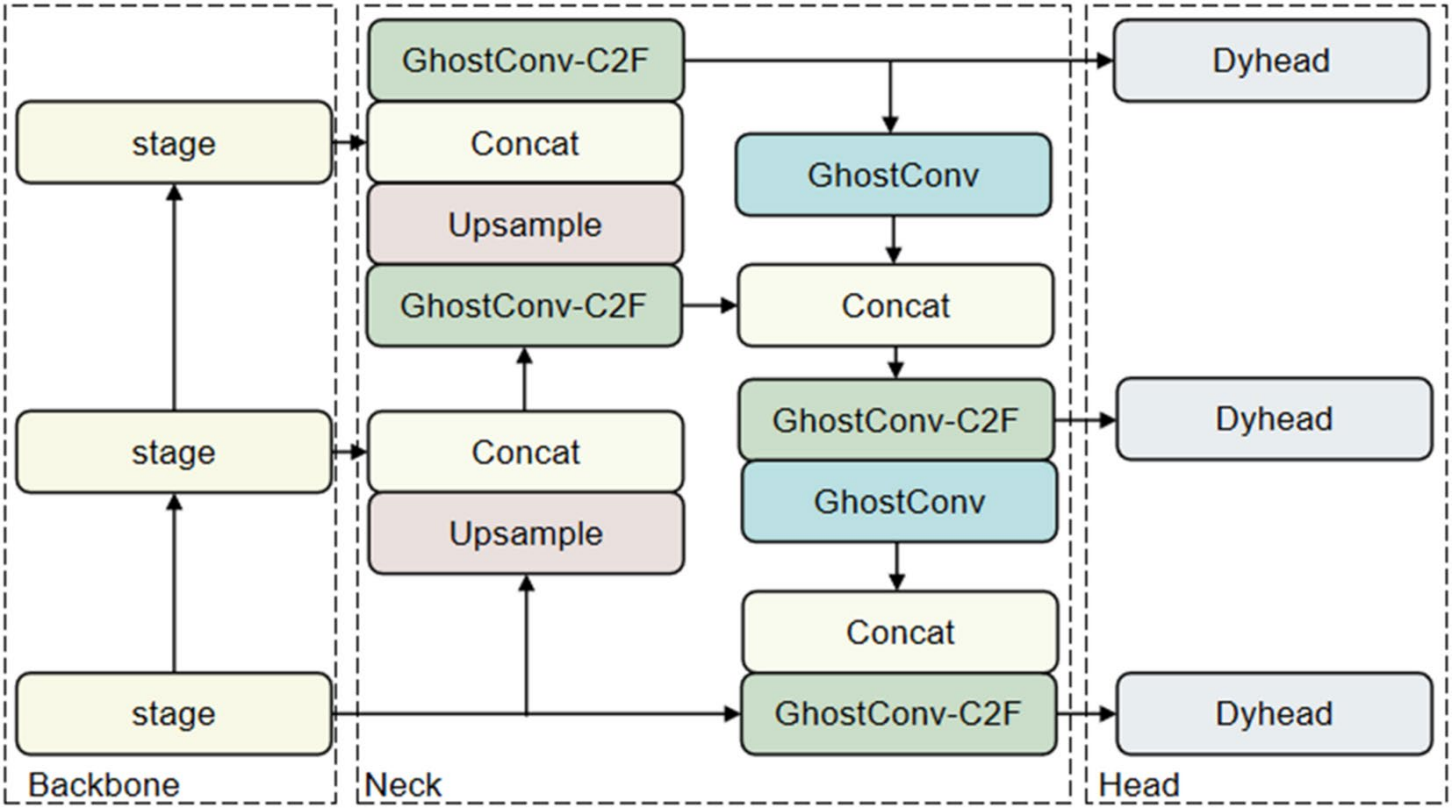
The neck network of NTRM-YOLO.

In order to make our model more lightweight for future use in non-tobacco related material robotic arms, we introduced the GhostConv model and GhostConv-C2F module in the Neck layer, forming a new Neck network of NTRM-YOLO, as shown in Fig. 8.

**Fig. 9:**
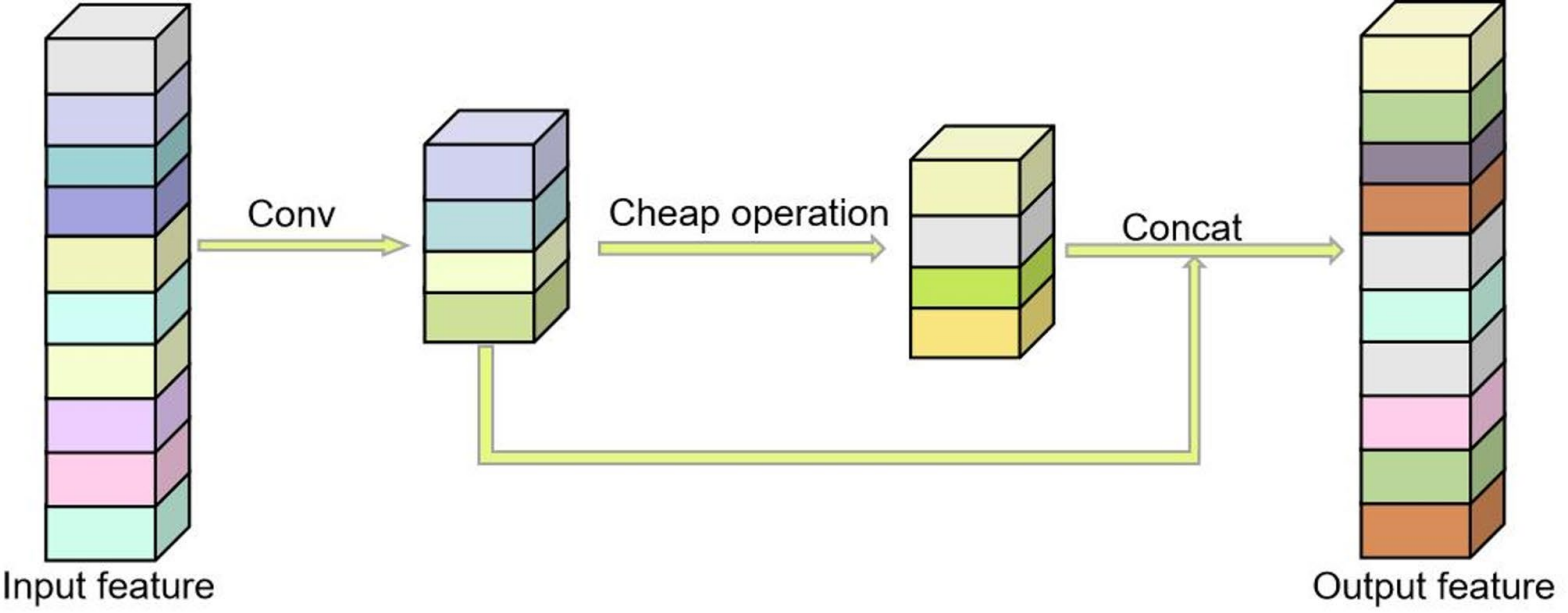
The structure of GhostConv.

GhostNet^42^ is a lightweight model proposed by Huawei Noah’s Ark Laboratory, and GhostConv is one of its convolutional modules. GhostConv abandons some conventional convolutions and uses linear transformations for feature extraction, which enhances the utilization of correlations and redundancies among features. The structure of GhostConv is depicted in Fig. 9. GhostConv initially employs a limited number of convolutional kernels to extract features and acquire part of the feature maps. Subsequently, another part of the feature map is computed via a lightweight linear transformation, known as the Cheap operation. Finally, concatenate these two feature maps into a complete feature map using the concat operation. This study substituted the Conv module with the GhostConv module in the neck network, thereby reducing the parameters without compromising model performance.

**Fig. 10:**
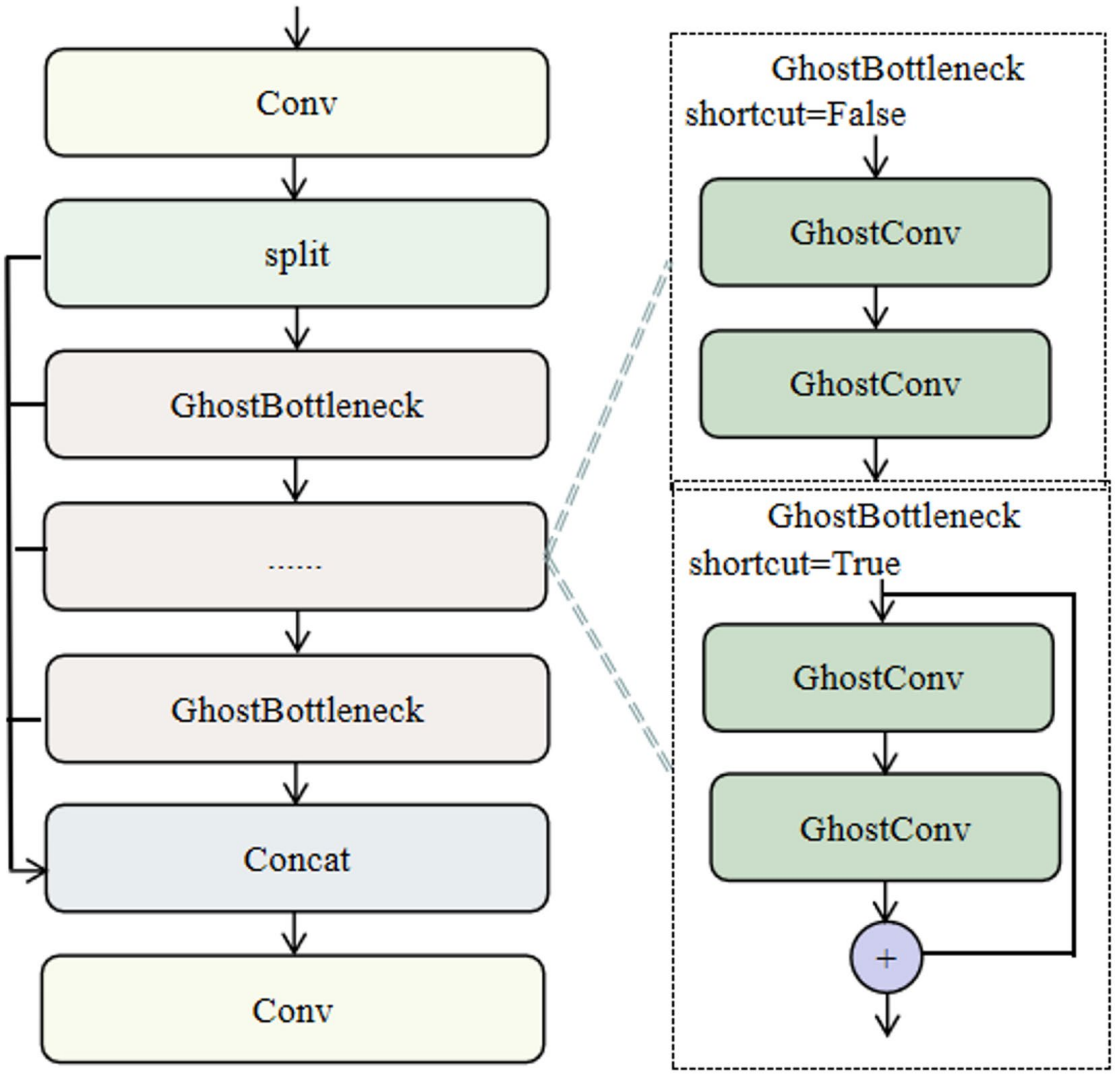
The structure of GhostConv-C2F.

To further diminish the model’s parameters, this study employed GhostConv in designing the GhostConv-C2F module, as shown in Fig. 10. Substituting the Bottleneck component in the original C2f module with GhostBottleneck and implementing GhostConv instead of Conv within GhostBottleneck can significantly diminish redundant computations inherent in conventional convolution, consequently lowering the parameter count without compromising feature extraction capabilities.

### The head network of NTRM-YOLO

**Fig. 11:**
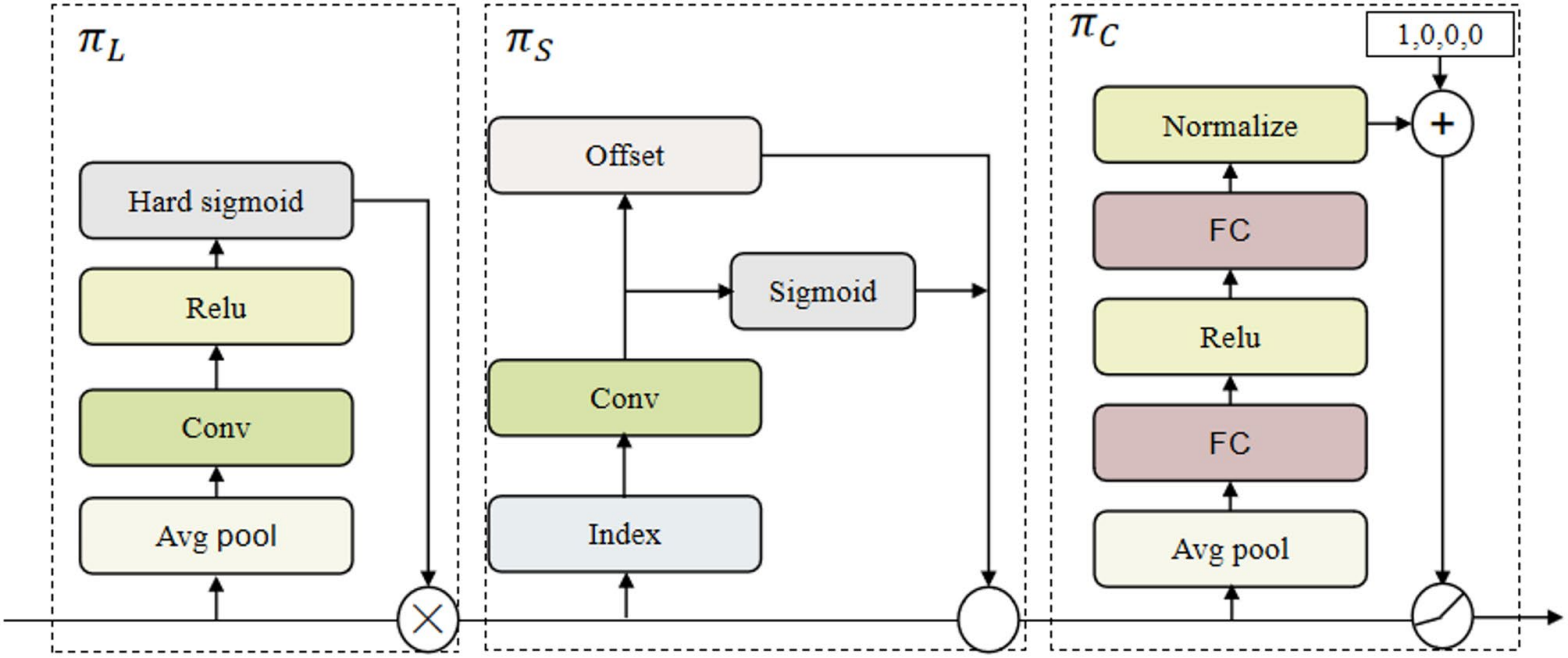
The structure of Dyhead: $\:{\:{\uppi\:}}_{\text{L}}$is scale-aware attention, $\:{\:{\uppi\:}}_{\text{S}}$is spatial-aware attention, $\:\text{a}\text{n}\text{d}{\:{\uppi\:}}_{\text{C}}$ is task-aware attention.

Within a tobacco sample, there may encounter one or more non-tobacco related materials typically intermixed with tobacco leaves, and easily obscured by the leaves. Furthermore, these non-tobacco related materials vary in size. Consequently, it is imperative to employ detection heads of varying scales to effectively identify non-tobacco related materials of diverse sizes during detection. Simultaneously, non-tobacco related materials within tobacco samples vary in position, with each instance exhibiting a distinct shape. Hence, the detection head must possess spatial awareness to effectively identify these non-tobacco related materials. Dyhead^43^ presents a dynamic head framework that leverages attention mechanisms to integrate the detection heads for various targets. Consequently, this study opts for Dyhead over the detection head in the original YOLOv8, the sturcture of Dyhead is depicted in Fig. 11.

Dyhead seamlessly incorporates scale awareness, spatial awareness, and task awareness into its framework. Initially, in the processing of non-tobacco related materials image features in the sequence dimension, the objective is to ascertain the relative significance among multiple semantic layers. Subsequently, features at appropriate levels are enhanced based on the size of non-tobacco related materials. The spatial perception attention module is designed to learn the distinctions among various non-tobacco related materials features based on their spatial positions within the image. The task perception attention module handles non-tobacco related materials feature data across channels, computes output differentials through the convolution of non-tobacco related materials features across various convolution kernels, and directs diverse feature channels to discern distinct types of non-tobacco related materials. Experimental results have shown that this approach significantly enhances the accuracy of model detection.

### Optimization of loss function

The YOLOv8 model employs a hybrid approach, combining CIoU and DFL loss methodologies for loss calculation. Specifically, DFL assesses the loss probability concerning bounding boxes and labels using cross-entropy format. CIoU computes the loss between the predicted box and the ground box. However, CIoU does not address the challenge of angle disparity between the ground and predicted boxes, potentially resulting in inaccurate regression predictions. To address this challenge, the study introduces the SIoU loss function proposed by Gevorgyan in 2022^44^. This loss function can calculate using the following formula:

1 $$\:{\text{L}\text{o}\text{s}\text{s}}_{\text{S}\text{I}\text{o}\text{U}}=1-\text{I}\text{o}\text{U}+\frac{{\Omega\:}+\Delta}{2}$$

Where, $\:{\Omega\:}$ represents the shape cost, $\Delta$ represents the distance loss, $\:{\Omega\:}$ and $\Delta$ can be calculated separately by the following formulas:

2 $$\:{\Omega\:}=\sum\:_{\text{t}=\text{w},\text{h}}{(1-{\text{e}}^{-{{\upomega\:}}_{\text{t}}})}^{{\uptheta\:}}$$

3 $$\Delta={\sum\:}_{\text{t}=\text{x},\text{y}}(1-{\text{e}}^{-{\upgamma\:}{{\uprho\:}}_{\text{t}}})$$

If the real box is denoted b^gt^ and the predicted box denoted as b, in Formula (2), $\:{{\upomega\:}}_{\text{w}}=\frac{|\text{w}-{\text{w}}^{\text{g}\text{t}}|}{\text{m}\text{a}\text{x}(\text{w},\:{\text{w}}^{\text{g}\text{t}})}$, $\:{{\upomega\:}}_{\text{h}}=\frac{|\text{h}-{\text{h}}^{\text{g}\text{t}}|}{\text{m}\text{a}\text{x}(\text{h},{\text{h}}^{\text{g}\text{t}})}$. In Formula (3), $\:{{\uprho\:}}_{\text{x}}={\left(\frac{{\text{b}}_{{\text{c}}_{\text{x}}}^{\text{g}\text{t}}-{\text{b}}_{{\text{c}}_{\text{x}}}}{{\text{c}}_{\text{w}}}\right)}^{2}$, $\:{{\uprho\:}}_{\text{y}}={\left(\frac{{\text{b}}_{{\text{c}}_{\text{y}}}^{\text{g}\text{t}}-{\text{b}}_{{\text{c}}_{\text{y}}}}{{\text{c}}_{\text{h}}}\right)}^{2},{\upgamma\:}$=2-$\:{\Lambda\:}$, $\:{\Lambda\:}\:$represents the angle cost, and $\:{\Lambda\:}$ an be calculated by formula (4):

4 $$\:{\Lambda\:}=1-2\text{*}{\text{s}\text{i}\text{n}}^{2}\left(\text{a}\text{r}\text{c}\text{s}\text{i}\text{n}\right(\text{x})-\frac{{\uppi\:}}{4})$$

Where, $\:\text{x}=\frac{{\text{c}}_{\text{h}}}{{\upsigma\:}}$, $\:{\text{c}}_{\text{h}}$ represents the distance in the y-direction between the center points of the ground box and the predicted box, $\:{\upsigma\:}$ represents the distance between the center points of the ground box and the predicted box.

## Experiment and results

### Experimental environment

For the sake of impartiality, in this study, all experiments were carried out on the same computer. The experimentation utilized the Linux 3.10.0 operating system, featuring an Intel Xeon Gold 6126 CPU, PyTorch 1.12.1, and Python environment version 3.9. In order to expedite the training process, this study employed two NVIDIA graphics cards. Furthermore, all experiments in this article were trained from scratch and did not utilize pre-trained weights. Some important hyperparameter settings during the training phase of this article are shown in Table 1.

**Table 1:** Training parameters.

Parameter	Value
Initial learning rate	0.01
Momentum	0.937
Batchsize	16
Weight_decay	0.0005
Optimizer	SGD
Device	0, 1

### Evaluation metrics

Our experiment is to train the model on the training set of NTRM dataset, and then evaluate the performance of the model on the NTRM test set. This article uses precision (P), recall (R), F1, mean average precision (mAP), parameters (Params) and floating point operations (FLOPs) to evaluate the detection performance. The respective calculation formulas are as follows.

5 $$\:\text{P}=\frac{\text{T}\text{P}}{\text{T}\text{P}+\text{F}\text{P}}\:$$

6 $$\:\text{R}=\frac{\text{T}\text{P}}{\text{T}\text{P}+\text{F}\text{N}}\:$$

7 $$\:\text{F}1=\frac{2\times\:\text{P}\times\:\text{R}}{\text{P}+\text{R}}$$

8 $$AP =\:{\int\:}_{0}^{1}\text{P}\left(\text{R}\right)\text{d}\text{R}$$

9 $$\:\text{m}\text{A}\text{P}=\frac{1}{\text{N}}\sum\:_{\text{i}=1}^{\text{N}}{\text{A}\text{P}}_{\text{i}}$$

Where, TP represents true positive, indicates that the instance is of a certain non-tobacco related material category, and the prediction is also the non-tobacco related material category. FP stands for false positive, indicates that the instance is not of a certain category of non-tobacco related materials, but the prediction is the non-tobacco related material category. FN denotes false negative, indicates that the instance is not a certain category of non-tobacco related materials, and the prediction is not the non-tobacco related material category, too. APi signifies the average precision of the i-th type of target. N is the number of types of non-tobacco related materials. mAP@50 indicates that the mean average precision at IoU 0.5.

### Results

**Fig. 12:**
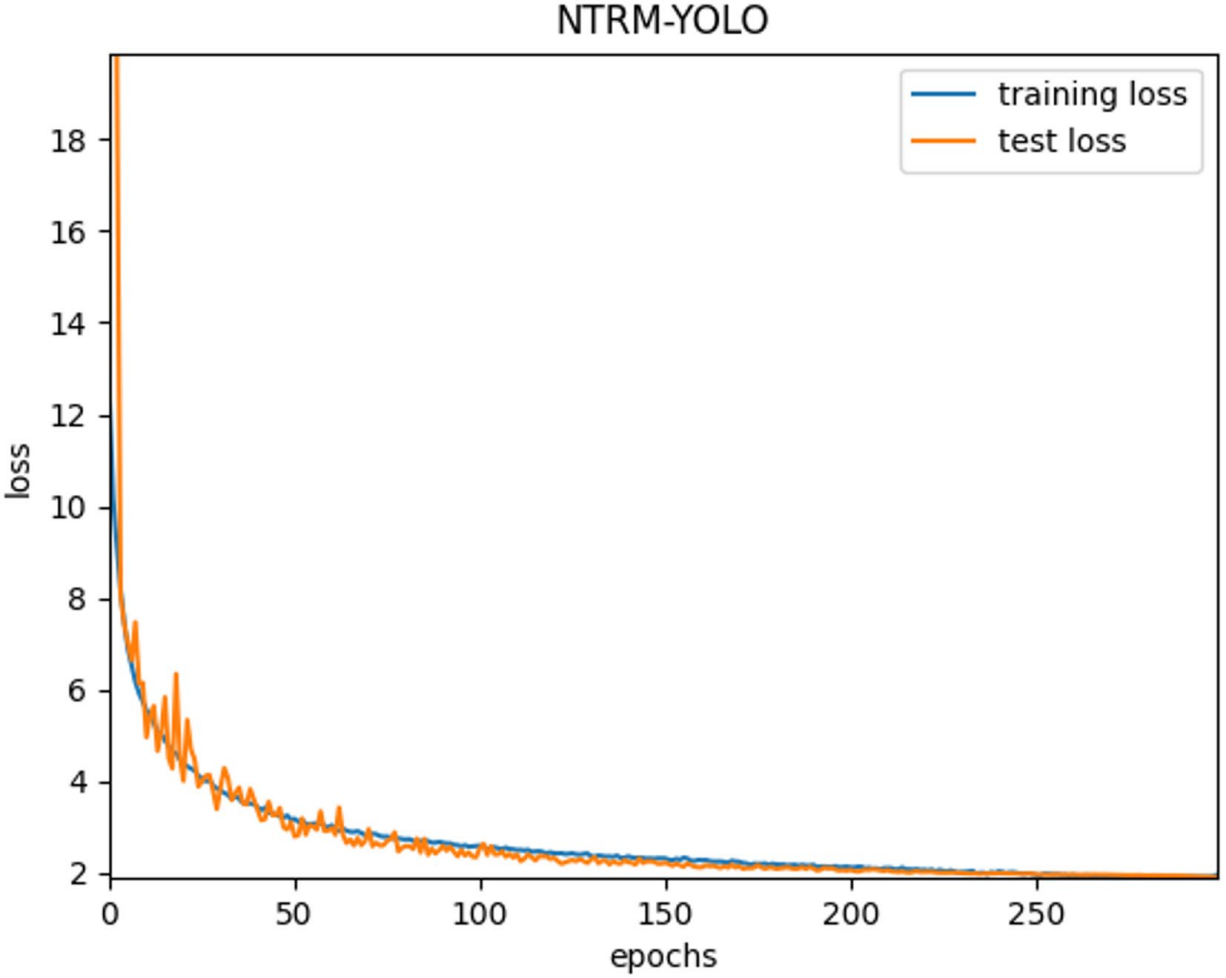
Loss function curves of baseline and NTRM-YOLO.

After the training of NTRM-YOLO on training set, the best model was selected for evaluation on the NTRM test set. Figure 12 (a) illustrates the loss function curves of NTRM-YOLO across both the training and test sets. The figure illustrates that the model achieves satisfactory convergence after 300 training iterations. Consequently, all subsequent experiments in this paper are conducted with 300 epochs.

#### Comparison with baseline

**Fig. 13:**
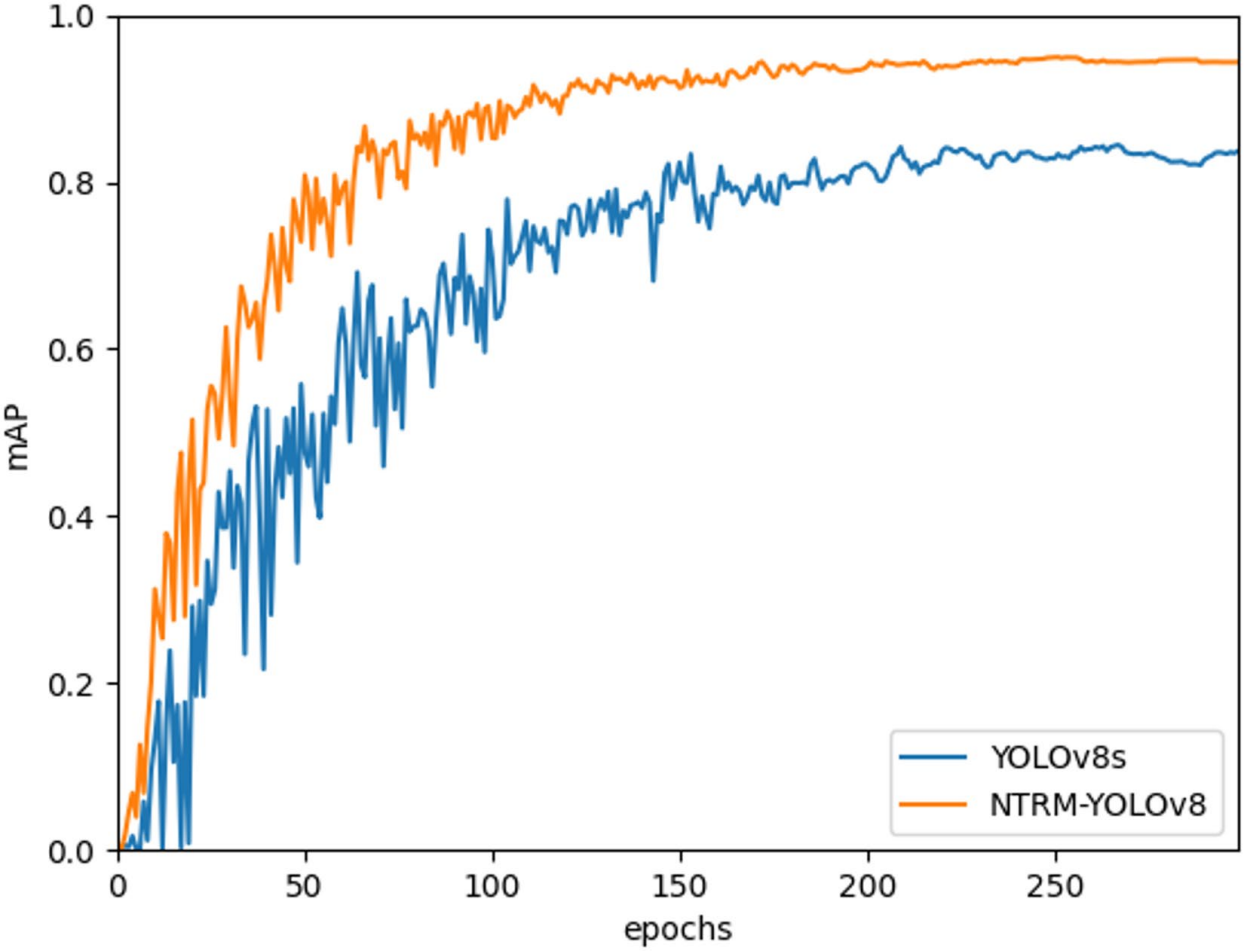
mAP@50 curves of baseline and NTRM-YOLO.

This study used YOLOv8s as the baseline model. Figure 13 is a graph showing the variation curves of mAP@50 for the baseline model and the NTRM-YOLO. In the figure, the orange mesh line represents our model. As can be seen from the figure, NTRM-YOLO generally achieves higher values than the baseline model.

Table 2 presents a comparative analysis between the baseline model and NTRM-YOLO in terms of P, R, F1, mAP@50, Params, and FLOPs. As depicted in the table, in terms of P, NTRM-YOLO is 3% higher than the baseline model. In terms of R, NTRM-YOLO is 0.8% higher than YOLOv8s. In terms of F1, NTRM-YOLO is 1.8% higher than YOLOv8s. Our model demonstrates strong performance in mAP@50, exhibiting a 2% point increase compared to YOLOv8s. Additionally, it reduced parameter by 1.2 MB, decreased FLOPs by 2.1G, and also saw a reduction in model size by 2.2 MB.

**Table 2:** Comparison between baseline and NTRM-YOLO.

Model	*P*	*R*	F1	mAP@50	Params(M)	FLOPs(G)
Baseline	0.937	0.899	0.918	0.936	11.2	28.8
NTRM-YOLO	0.967	0.907	0.936	0.956	10.0	26.7

**Fig. 14:**
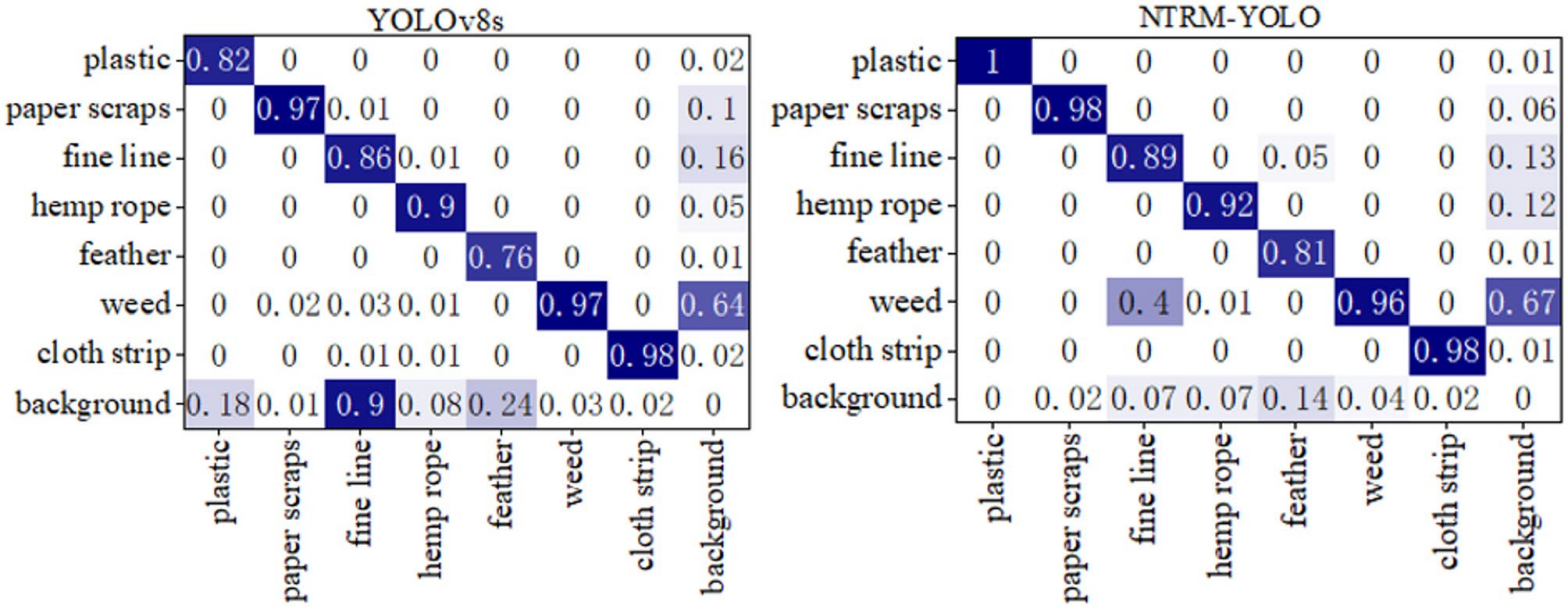
The confusion contrix of baseline and NTRM-YOLO.

Figure 14 displays the confusion matrix of the baseline model and NTRM-YOLO. In detecting various non-tobacco related materials, with the exception of feather class achieving an accuracy of 0.81, the accuracy for other types of non-tobacco related materials are notably high, reaching 100% accuracy for plastic. This substantiates the robust recognition capabilities of NTRM-YOLO for non-tobacco related materials. Upon comparison with YOLOv8s, it becomes apparent that NTRM-YOLO excels in detecting various non-tobacco related materials, showcasing superior performance compared to YOLOv8s.

In conclusion, NTRM-YOLO enhances accuracy while simultaneously reducing the parameters, showcasing the efficacy of our model in detecting non-tobacco related materials.

#### Comparison with different networks

To evaluate the performance of NTRM-YOLO, this study opted to compare it with several advanced models. Table 3 presents the experimental outcomes of models on the NTRM test set. In contrast to YOLOv3-tiny, NTRM-YOLO demonstrates enhancements in precision by 7.3% points, recall by 4.3% points, and F1 score by 5.7%. Moreover, its mAP@50 has improved by 5.8% points, while effectively reducing parameters by 2.1 MB. Although YOLOv5s, YOLOv6n, YOLOv7-tiny, and YOLOv8n possess smaller parameters than our model, but the values of mAP@50 of these models are 3.2% points, 13.7% points, 3.2% points, and 6.4% points less than our model. YOLOv5m, YOLOv6s, YOLOv7 and YOLOv8l, whether in mAP@50 or parameters, are not as good as NTRM-YOLO. Although YOLOv9-e was released in 2024, it still lags behind our model in terms of the evaluation metrics.

**Table 3:** Comparison results of various models.

Model	*P*	*R*	F1	mAP@50	Params(M)	FLOPs(G)
Faster-RCNN	0.553	0.857	0.672	0.602	41.7	18.7
Retinanet	0.615	0.884	0.725	0.792	36.5	15.5
YOLOv3-tiny	0.894	0.864	0.879	0.898	12.1	19.1
YOLOv5s	0.915	0.894	0.904	0.924	9.1	24.1
YOLOv5m	0.942	0.924	0.933	0.929	25.1	64.4
YOLOv6n	0.945	0.739	0.829	0.819	4.2	11.9
YOLOv6s	0.921	0.91	0.915	0.917	16.3	44.2
YOLOv7-tiny	0.878	0.917	0.897	0.924	6.0	13.2
YOLOv7	0.956	0.905	0.93	0.952	37.2	105.2
YOLOv8n	0.906	0.841	0.872	0.892	3.0	8.2
YOLOv8l	0.937	0.927	0.932	0.929	43.6	165.4
YOLOv9-e	0.947	0.9	0.923	0.945	69.4	244.9
Ours	0.967	0.907	0.936	0.956	10.0	26.7

We also compared it with the other object detection algorithm, the results are shown in Table 3. In contrast to Faster-RCNN, NTRM-YOLO’s mAP@50 improved by 35.4% points and reduced parameter count by 31.7 MB. The mAP@50 value of Retinanet is only 0.792, which is 16.4% less than our model. In conclusion, NTRM-YOLO demonstrates the best performance among all comparative models in this study.

**Fig. 15:**
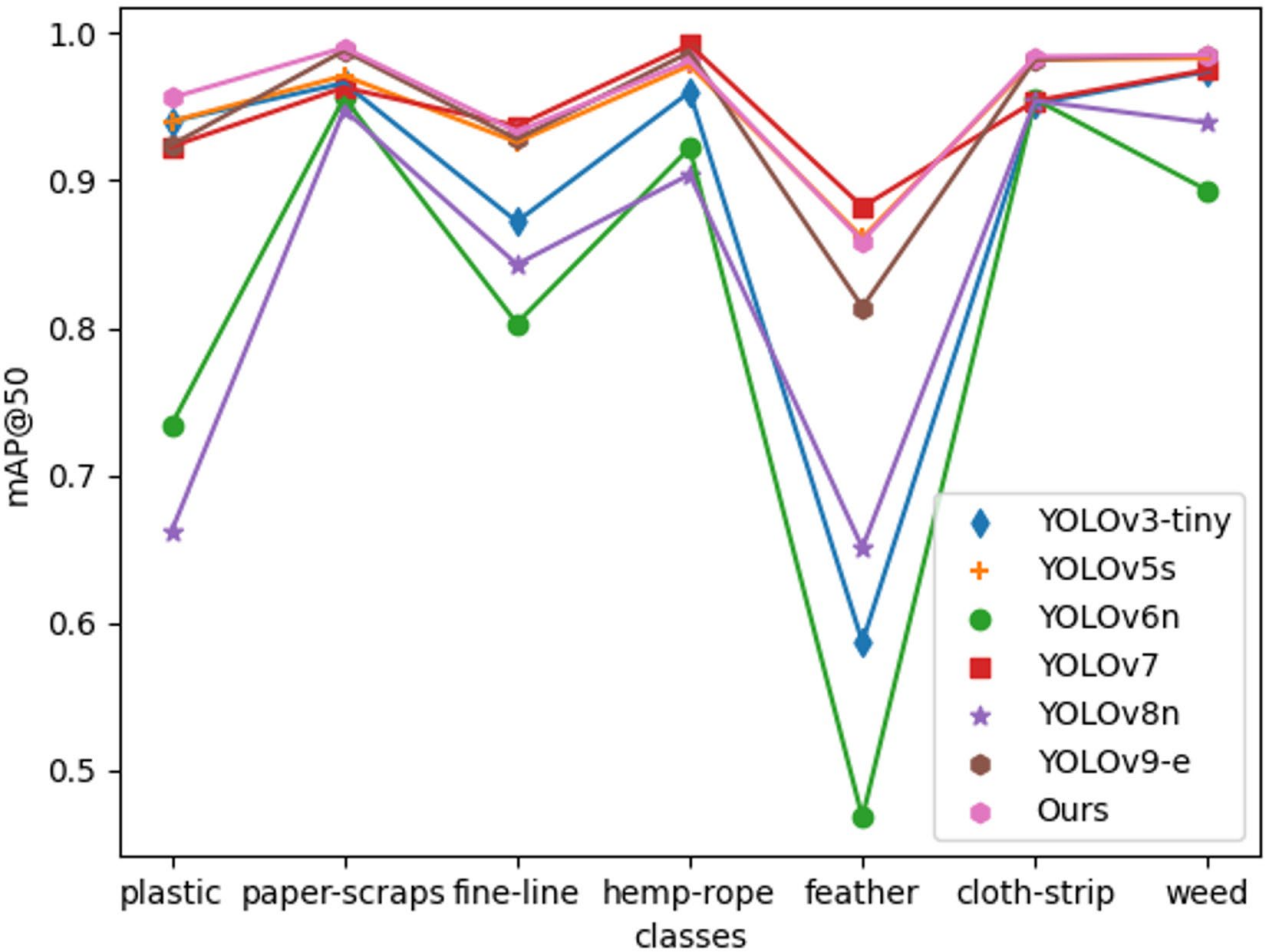
mAP@50 of different models for seven categories on NTRM test set.

Figure 15 showcases the value of mAP@50 of the selected models on the NTRM test set for the identification of seven types of non-tobacco related materials collected. We chosed YOLOv3-tiny, YOLOv5s, YOLOv6n, YOLOv7, YOLOv8n and YOLOv9-e to compare with our model. As depicted in the figure, our model achieves a plastic category detection rate of 95.6%, surpassing YOLOv3-tiny by 1.2%, YOLOv5s by 1.6%, YOLOv6n by 22.2%, YOLOv7 by 3.3%, YOLOv8n by 29.4%, and YOLOv9-e by 3.2%. In terms of paper-scraps category detection, our model achieves a performance level of 99%, surpassing YOLOv3-tiny by 2.4%, YOLOv5s by 1.9%, YOLOv6n by 3.4%, YOLOv7 by 2.7%, YOLOv8n by 4.2%, and YOLOv9-e by 0.2%. For the detection of fine-line category, our model attains a rate of 93.3%, exceeding YOLOv3-tiny by 6.1%, YOLOv5s by 0.7%, YOLOv6n by 13%, YOLOv8n by 9%, and YOLOv9-e by 1.5%. In the realm of hemp-rope category detection, our model attained the highest performance at 98.1% among all selected models. For the detection of feature category, our model achieves a commendable 85.9%, surpassing other models in performance. In terms of cloth-stip and weed, NTRM-YOLO also attained the highest performance. This analysis demonstrates that NTRM-YOLO achieves high performance in detecting diverse non-tobacco related materials, thereby validating the effectiveness of our model.

#### Ablation study

Ablation experiments were conducted to assess the enhancement of the model’s detection capabilities resulting from each proposed improvement module outlined in this study. Commencing with the YOLOv8s as the baseline model, we systematically refine it by incorporating various enhancement modules. Initially, we introduced the CPCA module to optimiz the backbone, followed by the integration of GhostConv and GhostConv-C2F. Subsequently, we adapt Dyhead, culminating in the optimization of the loss function. All experimental environment and hyperparameter are consistent, the results detailed in Table 4.

As depicted in Table 4, incorporating only the CPCA module resulted in a 1.3% point increase in mAP@50 on the NTRM test set. However, the parameters increased by 0.4 MB, the FLOPs increased by 1.5G. This suggests that the CPCA module filtered out irrelevant areas and heightened attention to valuable areas, thereby enhancing the effectiveness of model detection, albeit at the expense of increased Params and FLOPs. Building upon this foundation, substituting the Conv module with GhostConv resulted in a reduction of Params by 0.4 MB, signifying the effective diminution of the model’s parameters by the GhostConv module. Upon introducing GhostConv-C2F, the Params experienced a decrease of 0.9 MB. The modification of Dyhead resulted in an improvement of 2.2% points in mAP@50, accompanied by a decrease in Params of 0.3 MB. Ultimately, by adjusting the loss function to SIoU, the mAP@50 achieved to 95.6%, with no change observed in the Params and FLOPs. These experiments demonstrate that each enhancement module positively impacts the overall performance of NTRM-YOLO.

**Table 4:** Ablation Study results.

Model	*P*	*R*	F1	mAP@50	Params(M)	FLOPs(G)
Baseline	0.937	0.899	0.918	0.936	11.2	28.8
Optimized backbone	0.928	0.902	0.915	0.949	11.6	29.3
Optimized backbone + GhostConv	0.932	0.918	0.925	0.94	11.2	28.9
Optimized backbone + neck	0.915	0.931	0.922	0.931	10.3	27.0
Optimized backbone + neck + dyhead	0.963	0.901	0.931	0.953	10.0	26.7
Ours	0.967	0.907	0.936	0.956	10.0	26.7

#### Comparison of detection performance of different attention mechanisms

This study selected three attention mechanisms: SE^45^, CBAM^46^ and SimAM^47^ to ascertain the efficacy of the CPCA module. Due to these attention mechanisms exert minimal influence on the Params and Flops of the model, this section of the experiments only focuses on comparing the mAP@50. The experimental results were derived from the NTRM test set, as illustrated in Table 5. It is noteworthy that the model utilized in this section is exclusively derived by integrating an attention mechanism module into YOLOv8s. The table illustrates that among the four attention mechanisms, CPCA can accurately detect various non-tobacco related materials.

**Table 5:** Comparison of detection performance of different attention mechanisms on various NTRM.

Method	plastic	paper scarp	fine line	hemp rope	feather	weed	cloth strip
SE	0.891	0.969	0.939	0.981	0.85	0.984	0.982
SimAM	0.86	0.969	0.939	0.976	0.843	0.977	0.986
CBAM	0.804	0.975	0.901	0.984	0.672	0.981	0.983
This method	0.914	0.974	0.945	0.986	0.862	0.981	0.979

#### Comparison of detection performance of different loss function

**Fig. 16:**
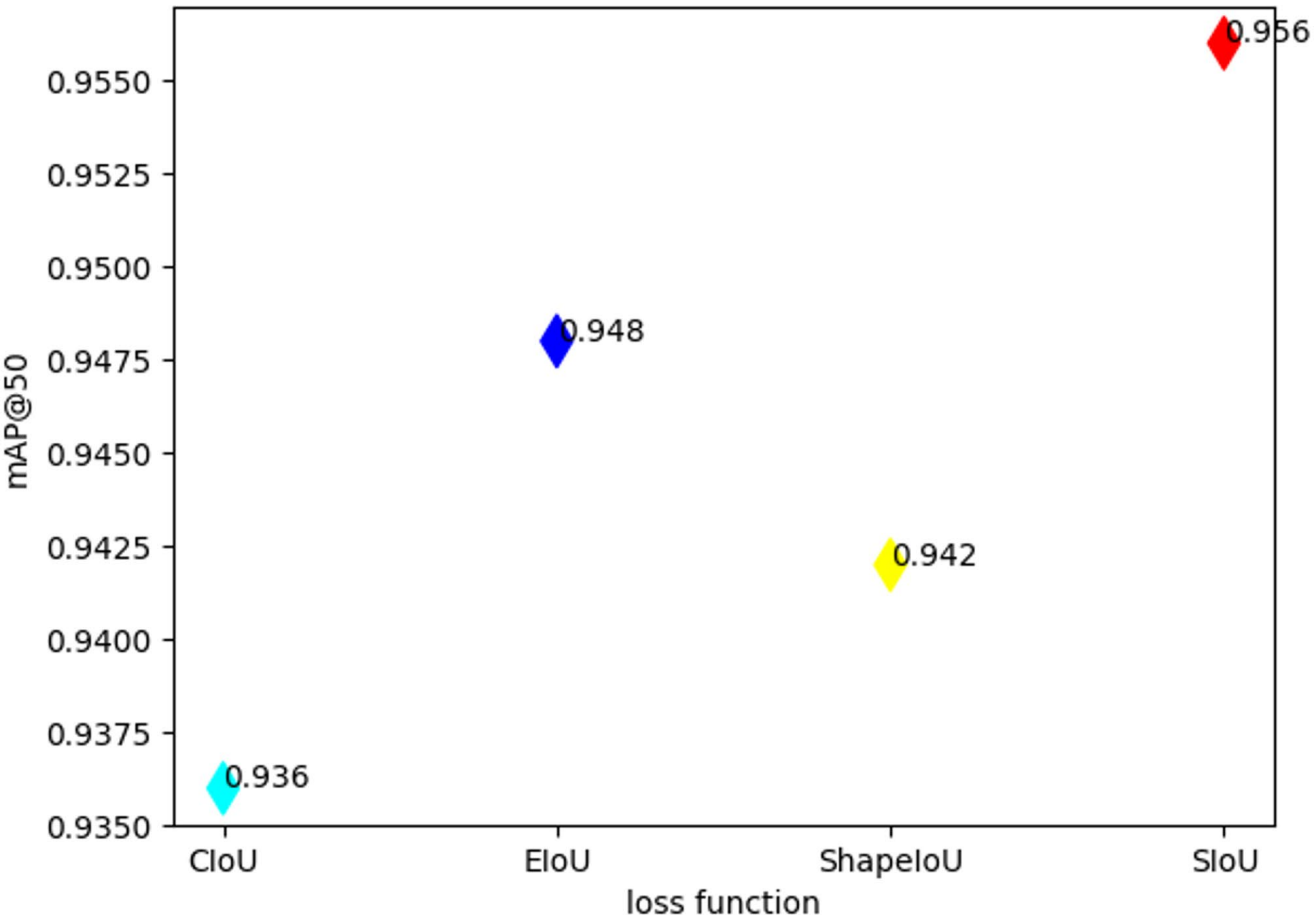
Obtained by different loss functions mAP@50 compare.

To validate the efficacy of the SIoU loss function, this section selected CIoU, EIoU and ShapeIoU for experiments under uniform condition. Similarly, this section of the experiments only focuses on comparing the mAP@50. As illustrated in Fig. 16, the SIoU achieved the highest mAP@50, which is 1.4%, 0.8%, 2.0% higher than ShapeIou, EIoU and CIoU, thus demonstrating the effectiveness of the SIoU loss function.

#### Visual analysis

**Fig. 17:**
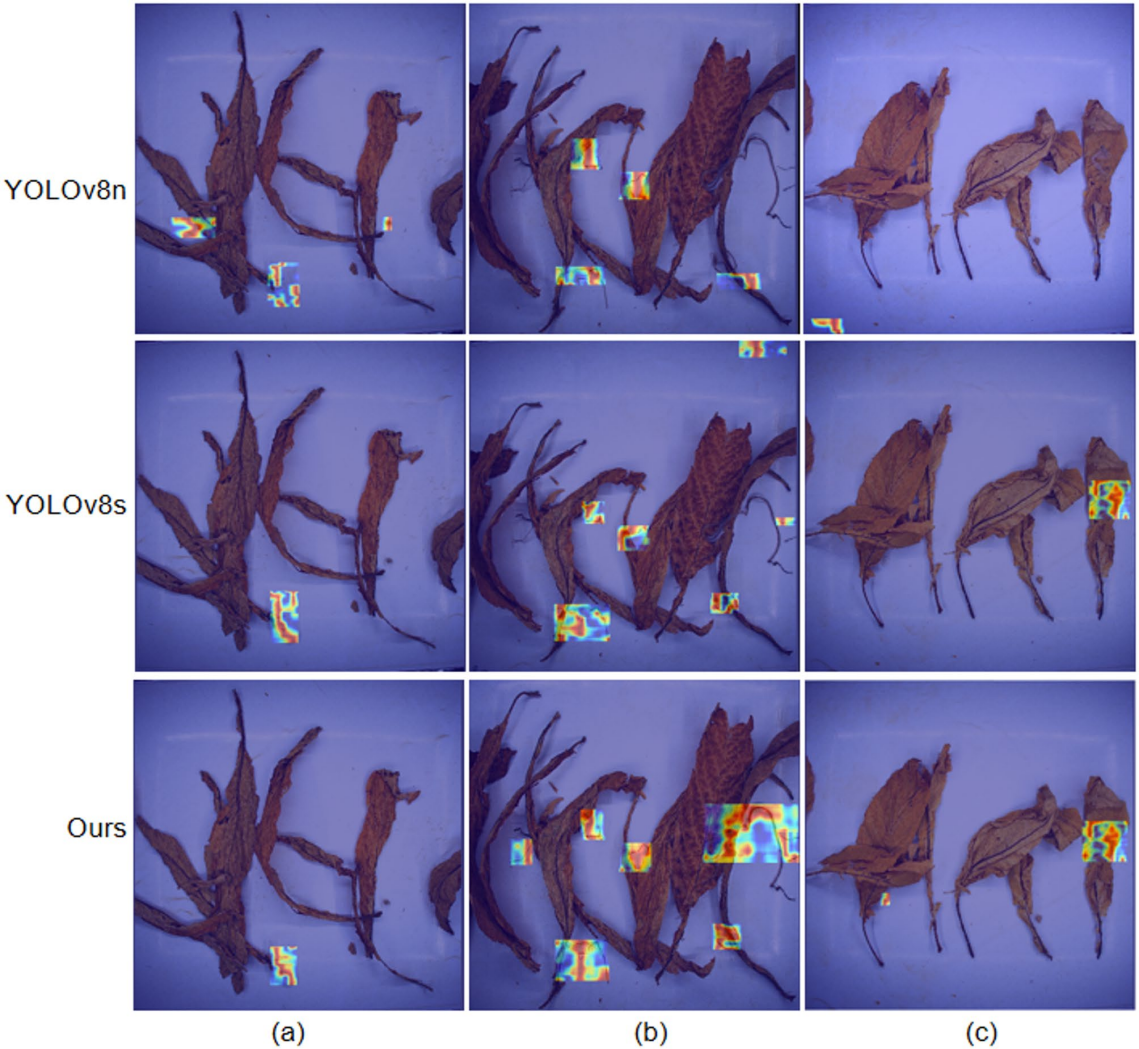
A visualization of YOLOv8n, YOLOv8s and Our model.

To visually ascertain the improvement afforded by the method proposed in this article in detecting non-tobacco related materials, multiple images were selected for comparison with YOLOv8n, YOLOv8s, and NTRM-YOLO models, as depicted in Fig. 17. In Fig. 17(a), YOLOv8n erroneously classified the tobacco leave as non-tobacco related material. YOLOv8s and NTRM-YOLO accurately detected the non-tobacco related material, but the feature map of NTRM-YOLO cover more foreground objects. In Fig. 17(b), YOLOv8n and YOLOv8s failed to detect some non-tobacco related materials, whereas the feature map of NTRM-YOLO cover all the non-tobacco related materials. In Fig. 17(c), YOLOv8n failed to detect any non-tobacco related materials, YOLOv8s failed to detect the non-tobacco related material that pressed by the tobacco leaves, whereas NTRM-YOLO accurately identified. It is evident that NTRM-YOLO can effectively detect non-tobacco-related materials.

## Conclusion

To tackle the challenge of detecting non-tobacco related materials, this article introduces the NTRM-YOLO method. This method incorporates the CPCA attention mechanism to construct a new backbone network, thereby enhancing the model’s capability for feature extraction and target perception, specifically for non-tobacco related materials. Furthermore, within the neck layer, the standard Conv module is replaced with the GhostConv module, and a novel GhostConv-C2F module is designed to replace the original C2F module. These modifications effectively reduce the model’s parameter count and computational complexity. Ultimately, by optimizing the detection head and loss function, the model’s performance in detecting non-tobacco related materials is significantly improved. The efficacy of this proposed approach is substantiated through a series of comparative and ablation experiments.

Further refinements to the methodology presented in this article are possible. Future research should consider the following aspects: (1) This study focuses solely on detecting seven types of non-tobacco related materials, using a limited number of collected samples. Enhancing the model’s universality is crucial for its further development. Future work aims to gather a larger dataset of images featuring diverse non-tobacco related materials and expand the scope to investigate a wider variety of these materials. (2)There is potential to increase the NTRM-YOLO model’s accuracy on the non-tobacco materials dataset. Subsequently, further optimization is needed to enable the model to handle more diverse types of non-tobacco materials. (3) While NTRM-YOLO demonstrates proficiency on the NTRM dataset collected in this study, its performance in alternative scenarios remains unverified empirically. It is therefore imperative to investigate the model’s applicability across a broader spectrum of situations in forthcoming research.

## Data Availability

The data used to support the findings of this study are available from the corresponding author upon request.
